# Life-Saving Emergency Adrenalectomy in a Pheochromocytoma Crisis with Cardiogenic Shock

**DOI:** 10.1155/2021/8848893

**Published:** 2021-03-18

**Authors:** Thalia Bekelaar, Gervais Nougon, Marc Peters, Frederic De Roeck, Steven Haine, Dirk Ysebaert, Maarten Spinhoven, Philippe G. Jorens, Rudi De Paep, Frederik Lahaye

**Affiliations:** ^1^Department of Cardiology, Antwerp University Hospital, University of Antwerp, Edegem, Belgium; ^2^Department of Emergency Medicine, Sint-Jozef Hospital, Malle, Belgium; ^3^Department of Hepatobiliary-Transplantation-Endocrine Surgery, Antwerp University Hospital, University of Antwerp, Edegem, Belgium; ^4^Department of Radiology, Antwerp University Hospital, University of Antwerp, Edegem, Belgium; ^5^Intensive Care Medicine, Antwerp University Hospital, University of Antwerp, Edegem, Belgium

## Abstract

Cardiogenic shock during a pheochromocytoma crisis is a life-threatening disorder. This case report illustrates a 49-year-old male with profound cardiogenic shock, extreme hemodynamic instability (systolic blood pressure ranging from 45 up to 290 mmHg in a cyclic pattern), and progressive multiple organ failure in the presence of a unilateral adrenal mass. Emergency adrenalectomy led to rapid hemodynamic stabilization. Histological investigation confirmed the diagnosis of pheochromocytoma. This case indicates that emergency adrenalectomy, although usually not considered first choice, is a valid option in cardiogenic shock and extremely fluctuating hemodynamics due to a pheochromcytoma-induced catecholamine storm.

## 1. Introduction

A pheochromocytoma crisis is an acute, life-threatening condition [1–3]. Excessive catecholamine release can cause hemodynamic instability, multiple organ failure, and cardiogenic shock. The primary underlying mechanism is activation of alpha-adrenergic receptors, which induces arterial vasoconstriction, reduced end-organ perfusion, and tissue ischemia [1]. The best treatment strategy during an acute crisis is still unclear, but hemodynamic stabilization prior to intervention is considered most appropriate. We present a case in whom emergency adrenalectomy of a (presumed) pheochromocytoma during cardiogenic shock led to a quick recovery.

## 2. Case Presentation

A 49-year-old male patient presented at the emergency room of the referring hospital with hematemesis and abdominal pain. The previous medical history included active smoking, epileptic seizures treated with lamotrigine, and an ischemic stroke treated with aspirin and simvastatin.

**Figure 1:**
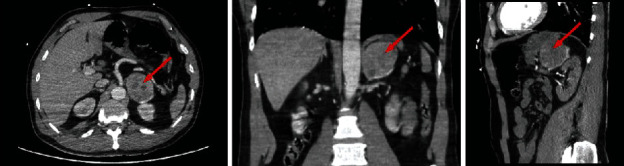
Abdominal computed tomography scan showed a left-sided adrenal mass of 5.7 cm (arrow).

Clinical examination showed a tender abdomen. His vital signs at initial evaluation were an oxygen saturation of 85% on room air, a blood pressure of 177/125 mmHg, pulse rate 127 bpm, body temperature 37.3°C, and Glasgow coma scale of 15/15. Arterial blood gas analysis revealed a pH of 7.25, with a paO_2_ of 52 mmHg, paCO_2_ of 33 mmHg, a marked elevated serum lactate 7.2 mmol/L (normal value < 2.2 mmol/L), and a low bicarbonate level 14 mmol/L (normal range 21–26 mmol/L). Due to progressive respiratory failure with hypoxemia and hypercapnia, the patient was intubated in the emergency room. Laboratory data showed an increased hemoglobin level of 18.8 g/dL (normal range 13.5-17.0 g/dL) and leukocytosis of 30.6 × 10^9^/L (normal range 4.3–9.6 × 10^9^/L) with a normal CRP (<5 mg/L). Acute renal failure with a creatinine of 2.5 mg/dL (normal range 0.7-1.3 mg/dL) was seen albeit normal electrolyte serum levels, accompanied by mildly deranged liver function tests, mildly elevated NT-pro-BNP (399 pg/mL; normal range 20-125 pg/mL), and markedly elevated high-sensitive troponin I level (2.490 ng/mL; normal range < 0.4 ng/mL). The electrocardiogram displayed sinus tachycardia with normal repolarization. Chest X-ray was compatible with pulmonary edema. Echocardiography showed a severe left ventricular dysfunction (ejection fraction of 25%) due to hypokinesia of the mid and basal segments, with preserved apical contractility. A Takotsubo syndrome was suspected. On the abdominal computerized tomography (CT) scan, which was performed because of the tender abdomen, a left-sided adrenal mass of 5.7 cm was seen (Figure 1).

**Figure 2:**
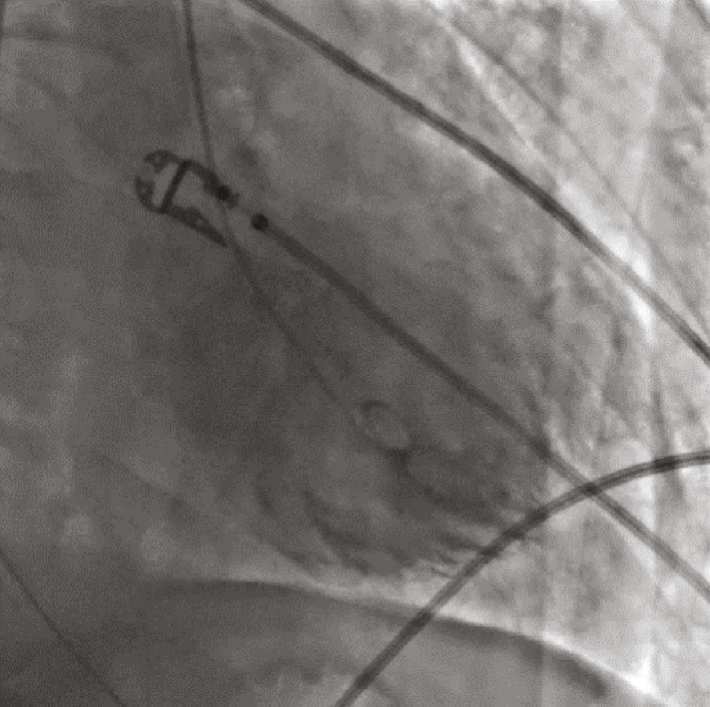
Left ventricular angiography during systole (RAO 24°/CRAN 8°). Severe hypokinetic midventricular segments compatible with an inverted Takotsubo syndrome (see also Supplementary Material: S1 video).

The patient was referred to our tertiary intensive care unit (ICU) for hemodynamic stabilization and further (cardiac) investigation. Urgent coronary angiography revealed triple vessel disease with significant stenoses in the midleft anterior descending coronary artery (LAD) and proximal left circumflex coronary artery (LCX) and a chronic total occlusion of the proximal RCA with collaterals from the left-sided coronary artery. Ventriculography was suggestive for a basal (reverse or inverted) Takotsubo syndrome, with severe hypokinetic midventricular segments (Figure 2, Svideo). Revascularization was not performed at that stage because of the inconsistency between the coronary lesions and the regional wall abnormalities and the severe hemodynamic instability, due to a suspected pheochromocytoma, possible requiring surgery in the future.

**Figure 3:**
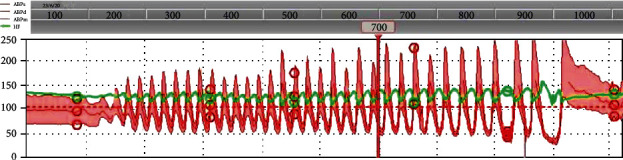
Invasive blood pressure monitoring. Cyclic fluctuations of blood pressure and heart rate with systolic blood pressures ranging from 45 mmHg up to 290 mmHg despite administration of alpha-blocking agent Phenoxybenzamine. Stabilization after emergency adrenalectomy. ABPs: arterial blood pressure systolic; ABPd: arterial blood pressure diastolic; ABPm: arterial blood pressure mean; HF: heart frequency.

The patient was deeply sedated and treated with the alpha-blocking agent phenoxybenzamine at high dose (20 mg b.i.d.) and an intra-aortic balloon pump. Nevertheless, he remained profoundly unstable with extreme blood pressure fluctuations (systolic blood pressure ranging from 45 mmHg to 290 mmHg) (Figure 3). Biochemically, progressive multiple organ failure was documented, with deterioration of the kidney and liver functions and a further rise in high-sensitive troponin I level to 49.821 ng/L (normal value < 45 ng/L). The placement of an extracorporeal life support (ECLS) was seriously considered, though not executed in view of the extreme hemodynamic instability.

As a catecholamine crisis was the most likely reason for the persistent hemodynamic instability, cardiogenic shock, and progressive multiple organ failure, an urgent open adrenalectomy was performed 21 hours after admission in the ICU; in the presence of board certified anesthesiologists, ICU physicians, and a skilled abdominal surgeon. Intraoperative rigorous fluid resuscitation was needed. After clamping of the adrenal vein and initiation of vasoactive drugs (adrenaline, noradrenaline, vasopressin, and phenylephrine), hemodynamic stabilization was rapidly achieved. Postoperatively, inotropes and vasopressors were continued. The IABP could be removed after 4 days. Due to further deterioration of the renal function, the patient received continuous venovenous hemofiltration during 7 days.

A 24-hour urine collection at admission showed extremely elevated levels of adrenaline (3001 *μ*g/g, normal value 1–44 *μ*g/g), noradrenaline (1600 *μ*g/g, normal value 9–112 *μ*g/g), metanephrine (3796 *μ*g/g, normal value 20–174 *μ*g/g), dopamine (435 *μ*g/g, normal value 30–350 *μ*g/g), and normetanephine (1210 *μ*g/g, normal value 48–452 *μ*g/g), compatible with massive catecholamine release by a pheochromocytoma. Histological investigation confirmed the presumed diagnosis of pheochromocytoma. A control echocardiography 7 days postoperatively showed a left ventricular ejection fraction recovery to 50%, further confirming the suspicion of left ventricular systolic dysfunction due to Takotsubo. Three weeks after surgery, coronary artery revascularization was performed with implantation of drug-eluting stents in the mid-LAD and proximal LCX stenosis. The patient was discharged from the ICU 16 days after admission and from the hospital after 24 days. At a follow up visit 2 weeks after discharge and now 3 months after the acute event, the patient was doing fine.

## 3. Discussion

The pathophysiological mechanism of cardiac dysfunction in a pheochromocytoma crisis is not fully understood but is mainly attributed to coronary artery vasospasm provoking myocardial ischemia and a direct toxic effect of the catecholamines on myocardial cells causing myocarditis and cardiomyopathy [3]. In most cases, left ventricular dysfunction presents as a typical or ‘inverted' Takotsubo cardiomyopathy [4], as was observed in our case. However, other left ventricular segments can also be affected due to individual differences in cardiac sympathetic innervation or distribution of adrenoreceptors [3]. In most patients, the cardiac dysfunction is fully reversible within a few days or weeks [5, 6]. This suggests that myocardial dysfunction is probably caused by myocardial stunning or metabolic anomaly rather than myocardial necrosis [7].

Mortality during a pheochromocytoma crisis remains high and is estimated at 15-30% [1, 2, 5]. Early diagnosis and appropriate management, including curative surgical resection, are considered essential. Based on retrospective studies, preoperative stabilization with medical treatment and fluid resuscitation is recommended, as it is believed to reduce morbidity and mortality due to hemodynamic stabilization [8, 9]. Pretreatment for 7 to 14 days before surgery is advised [9]. However, in case of an acute catecholaminergic crisis, optimal timing of surgery remains controversial as evidence from randomized controlled clinical trials is lacking [10].

Alpha-blocking agents, e.g., phenoxybenzamine or phentolamin, are the first choice therapy but not always instantly available [1]. Alternatively, calcium channel blockers can be administered, although profound hypotension in cardiogenic shock might be a limiting factor. Since beta-blockade could induce worsening of the hemodynamic status, it should only be used after prior initiation of alpha-blockade [1]. Add-on treatment with metyrosine to inhibit catecholamine synthesis can be considered [9].

In patients with a catecholaminergic crisis and cardiogenic shock, initiation of medical therapy can be challenging and organ-specific mechanical support is often needed. As represented in a multicenter retrospective study by Sauneuf et al., patients often require mechanical ventilation (85%), circulatory support (vasoactive drugs in 68% and extracorporeal membrane oxygenation (ECMO) in 41%), and renal replacement therapy (24%) [5]. Out of a pathophysiological perspective, circulatory support with inotropes or vasopressors could be associated with adverse effects on the heart and other end organs. Therefore, mechanical circulatory support, including ECLS, is increasingly used in patients with cardiogenic shock or refractory arrest to ensure adequate organ perfusion [11]. Unfortunately, ECLS-related complications (such as bleeding, infection, ischemic stroke, or lower limb ischemia) are frequent [5]. Furthermore, mechanical support systems might experience technical difficulties in patients with extreme fluctuating blood pressure, as was observed in our case.

In order to break the vicious circle of catecholamine excess and progressive organ failure, early surgical resection of a pheochromocytoma can be considered. Although a cohort study and review literature by Scholten et al. suggest that urgent surgery is associated with increased morbidity [8], these findings were biased since patients undergoing elective surgery were more stable at baseline. Also, when limiting their literature review to cases after 1990, mortality was only 1 in 18 cases (6%), which compares to the expected mortality for any severe crisis. More recent case reports and cohort studies showed no increased mortality in patients treated with emergency surgery [2, 5, 12, 13]. In a German cohort study by Riester et al., 2 out of 15 patients with life-threatening catecholamine crisis needed emergency resection because of cardiorespiratory instability and both patients survived [2]. In a French cohort study by Sauneuf et al., 5 of 34 patients underwent urgent surgery and all five were discharged alive [5].

In selected patients with extreme hemodynamic fluctuations, urgent adrenalectomy is the only option to achieve (rapid) hemodynamic stabilization, as is illustrated by our case [1, 2]. Of course, this delicate operation needs to be performed by a skilled multidisciplinary team with rigorous blood pressure monitoring and careful surgical handling of the tissue to prevent massive catecholamine release during surgical manipulation [10].

## 4. Conclusion

The first presentation of a pheochromocytoma can be a severe catecholaminergic crisis with hemodynamic instability, Takotsubo syndrome, cardiogenic shock, and multiple organ failure, as is illustrated in this case. Curative treatment consists of complete surgical resection. Preoperative medical management using alpha-blocking agents is recommended. Nevertheless, in persistently unstable patients with rapid deterioration, emergency resection with complete removal of the pheochromocytoma might be life-saving.

## Data Availability

Data are available if appropriate.

## Supplementary Materials

Supplementary 1 Table S1: timeline according to the CARE principles.

Supplementary 2 Video S1: left ventricular angiography with severe hypokinetic mid-ventricular segments compatible with an inverted Takotsubo syndrome.
