# Supplementary material for: Life-Saving Emergency Adrenalectomy in a Pheochromocytoma Crisis with Cardiogenic Shock
Source: Case Rep Cardiol. 2021 Mar 18;2021:8848893. doi: 10.1155/2021/8848893 (PMC7996044; doi:10.1155/2021/8848893)
Supplement: Supplementary 1 — Table S1: timeline according to the CARE principles. [file 8848893.f1.docx]

# Supplementary material

# Timeline according to the CARE-guidelines.

| Day 0 | Admission in emergency department with hematemesis and abdominal pain. Intubation due to respiratory distress.  Diagnosis: cardiogenic shock, multiple organ failure and intra-abdominal mass |
| --- | --- |
| Day 0 – 3h 30min | Transfer to Intensive Care Unit (ICU) in tertiary care hospital |
| Day 0 – 5h | Coronary angiography: triple vessel disease  Left ventricular angiography: inverted takotsubo cardiomyopathy  Implantation of intra-aortic balloon pump (IABP) |
| Day 0 – 14h | Hemodynamic instability – start alpha-blocking agent Phenoxybenzamine |
| Day 0 – 21h | Emergency open adrenalectomy |
| Day 3-10 | Continuous veno-venous hemofiltration |
| Day 4 | Removal IABP |
| Day 16 | ICU discharge |
| Day 22 | Coronary revascularization |
| Day 24 | Hospital discharge |
